# Nrf2 Activation Protects Against Organic Dust and Hydrogen Sulfide Exposure Induced Epithelial Barrier Loss and *K. pneumoniae* Invasion

**DOI:** 10.3389/fcimb.2022.848773

**Published:** 2022-04-19

**Authors:** Denusha Shrestha, Nyzil Massey, Sanjana Mahadev Bhat, Tomislav Jelesijević, Orhan Sahin, Qijing Zhang, Kristina L. Bailey, Jill A. Poole, Chandrashekhar Charavaryamath

**Affiliations:** ^1^ Department of Biomedical Sciences, Iowa State University, Ames, IA, United States; ^2^ Immunobiology Interdepartmental Graduate Program, Iowa State University, Ames, IA, United States; ^3^ Department of Comparative Biomedical Sciences, Louisiana State University, Baton Rouge, LA, United States; ^4^ Department of Veterinary Diagnostic and Production Animal Medicine, Iowa State University, Ames, IA, United States; ^5^ Veterinary Microbiology and Preventive Medicine, Iowa State University, Ames, IA, United States; ^6^ Department of Internal Medicine, Division of Pulmonary, Critical Care and Sleep Medicine, University of Nebraska Medical Center, Omaha, NE, United States; ^7^ Department of Medicine, University of Nebraska Medical Center, Omaha, NE, United States

**Keywords:** organic dust, Nrf2, RTA-408, H_2_S, *K. pneumoniae*

## Abstract

Agriculture workers report various respiratory symptoms owing to occupational exposure to organic dust (OD) and various gases. Previously, we demonstrated that pre-exposure to hydrogen sulfide (H_2_S) alters the host response to OD and induces oxidative stress. Nrf2 is a master-regulator of host antioxidant response and exposures to toxicants is known to reduce Nrf2 activity. The OD exposure-induced lung inflammation is known to increase susceptibility to a secondary microbial infection. We tested the hypothesis that repeated exposure to OD or H_2_S leads to loss of Nrf2, loss of epithelial cell integrity and that activation of Nrf2 rescues this epithelial barrier dysfunction. Primary normal human bronchial epithelial (NHBE) cells or mouse precision cut-lung slices (PCLS) were treated with media, swine confinement facility organic dust extract (ODE) or H_2_S or ODE+H_2_S for one or five days. Cells were also pretreated with vehicle control (DMSO) or RTA-408, a Nrf2 activator. Acute exposure to H_2_S and ODE+H_2_S altered the cell morphology, decreased the viability as per the MTT assay, and reduced the Nrf2 expression as well as increased the keap1 levels in NHBE cells. Repeated exposure to ODE or H_2_S or ODE+H_2_S induced oxidative stress and cytokine production, decreased tight junction protein occludin and cytoskeletal protein ezrin expression, disrupted epithelial integrity and resulted in increased *Klebsiella pneumoniae* invasion. RTA-408 (pharmacological activator of Nrf2) activated Nrf2 by decreasing keap1 levels and reduced ODE+H_2_S-induced changes including reversing loss of barrier integrity, inflammatory cytokine production and microbial invasion in PCLS but not in NHBE cell model. We conclude that Nrf2 activation has a partial protective function against ODE and H_2_S.

## Introduction

Worldwide agriculture sector employs an estimated 1.3 billion people, and agriculture workers are exposed to a variety of occupational hazards (reviewed in (Sethi et al., 2017; International Labor Organization, 2018). Among these, contaminants within the animal feeding operations are known to negatively impact the health of workers. The chief contaminants within the animal feeding operations settings include airborne organic dust, gases such as hydrogen sulfide (H_2_S) and other volatile organic compounds (Iowa State University and University of Iowa, 2002; Ni et al., 2012). Workers in the animal production settings are persistently exposed to airborne organic dust containing microbes and microbial products as well as gases such as H_2_S (Iowa State University and University of Iowa, 2002; Shrestha et al., 2021). Exposed individuals report both acute and chronic respiratory symptoms such as coughing, sneezing, bronchitis, chest-tightness, asthma and asthma-like symptoms, mucus membrane irritation, chronic obstructive pulmonary disease (COPD), with a decline in lung function (Sahlander et al., 2012; Sethi et al., 2017; Nordgren and Charavaryamath, 2018).

H_2_S gas is a known respiratory tract irritant [reviewed in (Zhang et al., 2021)]. Therefore, understanding how a combined exposure to organic dust and H_2_S would alter homeostasis of respiratory system is important. Our recent work (Shrestha et al., 2021) using a mouse model showed that a single pre-exposure to H_2_S altered the host response to organic dust. Notably, H_2_S pre-exposure or H_2_S+ODE exposure decreased cell viability, increased the transcripts of *tlr2* and *tlr4* as well as oxidative stress and inflammation markers. Further, H_2_S exposure decreased the transcript of *claudin1* to indicate a possible effect of the epithelial integrity. Organic dust extracts (ODE) from animal feeding operations induce a profound inflammatory response both *in vitro* and *in vivo* with several prior studies demonstrating strong roles for Toll-like receptor (TLR) and MyD88 signaling pathways (Charavaryamath et al., 2005; Charavaryamath et al., 2008; Charavaryamath et al., 2008; Poole et al., 2015). In airway epithelial cells, these exposures induce pro-inflammatory cytokine release, neutrophil influx, mucus metaplasia, alter tight junction expressions, disrupt cellular migration, and slow ciliary beat frequency (Wyatt et al., 2008; Bhat et al., 2019; Johnson et al., 2020; Shrestha et al., 2021). It has also been demonstrated that CO_2_ concentrations differentially effect the magnitude of inflammatory responses to ODE and various TLR ligands (Schneberger et al., 2015; Schneberger et al., 2021). Other occupational toxicants including particulate matter (PM) exposure result in increases in *Pseudomonas aeruginosa* infectivity due to disruption of airway epithelial barrier (Liu et al., 2019). We recently demonstrated that ODE and H_2_S exposure induced Nuclear factor-erythroid factor 2-related factor 2 (*Nrf2*) gene expression in monocytes and airway epithelial cells with associated decreased cell viability and increased reactive nitrogen species production (Shrestha et al., 2021). However, it is not known whether Nrf2 activity can be targeted to reverse adverse effects and whether exposure-induced airway epithelial cell dysfunction is associated with enhanced infection susceptibility to inform future strategies.

H_2_S and organic dust exposures induce an overwhelming oxidative stress response. Host cells respond to the oxidative stress *via* activation of the Nrf2 pathway (Shrestha et al., 2021). In the absence of an effective antioxidant response, unchecked release of reactive oxygen species (ROS), reactive nitrogen species (RNS) and inflammatory cytokines would result in lung homeostasis imbalance. Nrf2, together with Kelch-like ECH-associated protein 1 (Keap1), acts as a two-component system to tightly maintain the baseline and stimulated antioxidant responses (Yamamoto et al., 2018). Our previous data from the mouse model showed that an exposure to barn environment containing organic dust and gases induced airway epithelial damage (Charavaryamath et al., 2008). Further, our recent work (Shrestha et al., 2021) confirmed that H_2_S and ODE exposures induce oxidative stress, reduce the transcript of *claudin1* corresponding to the tight junction protein. These results warrant an investigation into the mechanisms of oxidative stress induced epithelial damage including whether exposures compromise epithelial barrier. Several toxic exposures and or diseases affect the Nrf2 activity and activation of Nrf2 using pharmacological or genetic tools appears beneficial (Tharakan et al., 2016; Zhao et al., 2017).

We have previously shown that organic dust exposure followed by a secondary microbial (LPS) challenge induced a more robust lung inflammation (Charavaryamath et al., 2008) *via* unknown mechanisms. Previous work has shown that exposure to swine production units containing organic dust and H_2_S induces airway epithelial damage (Charavaryamath et al., 2008). ODE is known to induce alterations in several tight junction components including occludin protein (Johnson et al., 2020). However, the effect of simultaneous exposure to organic dust and H_2_S on the airway epithelial tight junction proteins is unknown. Low grade H_2_S exposure has shown to be protective against the epithelial barrier disruption caused by cigarette smoke (Guan et al., 2020). Our published data showed that a single pre-exposure to H_2_S modulated the innate immune response in the lungs to ODE (Shrestha et al., 2021). Nevertheless, similar to the real-world occupational exposures, repeated exposures to H_2_S and organic dust are important in understanding the impacts on airway epithelial integrity.

In the current study, we tested a hypothesis that repeated exposure to organic dust or H_2_S leads to loss of Nrf2, loss of epithelial integrity and that activation of Nrf2 rescues the epithelial barrier dysfunction. Our data shows that Nrf2 activation rescues the organic dust and H_2_S exposure induced epithelial barrier dysfunction and decreased the bacterial invasion *via* attenuating the oxidative stress, inflammation markers and protecting tight junction proteins.

## Methods

### Cells, Biological and Chemical Reagents

De-identified Normal human bronchial epithelial (NHBE) cells were received through Dr. Kristina L. Bailey MD (University of Nebraska Medical Center, Omaha, NE) under an approved institutional review board protocol (UNMC IRB#318-09-NH). These NHBE cells were isolated from the human donors through the Live on Nebraska, an organ and tissue donation program. All the donors were deceased and informed consent for research was obtained by the Live on Nebraska from the next of the kin. These de-identified NHBE cells were deemed exempt from the Institutional Review Board (IRB) approval at the Iowa State University. However, these cells were received and used in our experiments under an approved Institutional Biosafety Committee (IBC) protocol (IBC# 19-004). NHBE cells were cultured in Pneumacult expansion media (STEMCELL Technologies, Vancouver, BC, Canada) supplemented with 100 U/mL of penicillin/streptomycin (Gibco) and 2 µg/mL of amphotericin B (Sigma) in a humidified chamber with 5% CO_2_ at 37˚C. Sodium hydrosulfide hydrate (NaSH, H_2_S donor in solution) and FITC dextran dye (average mol. wt. 4000) were procured from Sigma-Aldrich whereas RTA-408 (NRF2 activator) was obtained from MedChemExpress USA.

### Preparation of Organic Dust Extract (ODE)

Collection and handling of organic dust (OD) and preparation of a sterile organic dust extract (ODE) were performed as per an approved protocol from the Institutional Biosafety Committee (IBC protocol# 19-004) of the Iowa State University. Settled swine barn dust samples (n=7, representing organic dust) were collected from various swine production units into sealed bags with a desiccant and transported on ice to the laboratory. ODE samples were prepared as per a published manuscript (Romberger et al., 2002). Briefly, dust samples were weighed, and for every gram of dust, 10 mL of Hank’s balanced salt solution without calcium (Gibco) was added and allowed to stir at room temperature for an hour. The mixture was centrifuged (1365 g, 4°C) for 20 minutes, supernatant collected, and the pellet was discarded. The supernatant was centrifuged again with the same conditions, the pellet discarded and recovered supernatant was filtered using a 0.22 µm filter and stored at -80° C until used. This stock was considered 100% and diluted in cell culture medium to prepare a 0.5-1% (v/v) solution for use in experiments. We quantified the LPS concentration in these samples (1.198 ± 0.2333 EU/mL) and a pooled sample of ODE representing all the seven samples was used in our experiments.

### Preparation of Murine Precision Cut Lung Slices (PCLS)

All the animal breeding and experiments were performed under approved protocols from the institutional animal care and use committee (IACUC) at the Iowa State University, Ames, IA. Under approved protocols (IACUC-19-165 and IACUC-19-076), we procured breeding pairs of C57BL/6 wild type mice (The Jackson Laboratory, Bar Harbor, ME) and paired the males and females. All the animals had access to standard laboratory animal diet and drinking water *ad libitum* with adequate social enrichment and 12 h light/12 h dark cycles. The pups born out of the breeding pairs were weaned at 21 days of age and then housed separately. Mice aged 6-8 weeks (males or females) were euthanized by administering CO_2_ from a pressure cylinder as per the American Veterinary Medical Association (AVMA) Guidelines for Euthanasia of Animals. Following euthanasia, the trachea was exposed and using a 20-gauge needle, 0.8-1.0 mL of low-melting agarose (2%, prepared in 1X HBSS) was slowly instilled into the lungs. Next, whole carcass was placed at 4°C for about 20-30 minutes to allow solidification of the agarose within the lungs. The trachea-lung pluck was then carefully removed from the carcass taking care not to puncture the lungs and processed to prepare 300 µm thick sections using a compresstome (VF-300 Microtome, Precisionary Instruments Inc.). The precision cut lung slices were collected and cultured in DMEM media supplemented with 100 U/mL of penicillin/streptomycin (Gibco) and 2 µg/mL of amphotericin B (Sigma) in a humidified chamber with 5% CO_2_ at 37˚C. To remove the agarose, the media was initially changed every half an hour in the first two hours, every hour for the next two hours and finally, the media was changed every 24 hours until lung slices were used in the experiments. The viability of the PCLS was confirmed by the MTT assay (Akram et al., 2019) and observation of cilia beating under a microscope.

### Experimental Design

NHBE cells cultured between 2-3 passages were used for this study. When cells reached a 70-80% confluence, they were treated with either medium (control), medium with 0.5% ODE or 10 ppm H_2_S or both ODE and H_2_S together for 6 hours daily for 5 days (**Table 1**). We have previously used sodium hydrosulfide (NaSH) as a H_2_S donor in aqueous solution (Shrestha et al., 2021) and the amount of NaSH required to release 10 ppm H_2_S was titrated in separate pilot studies (data now shown). Murine lung slices (4-5/well) placed in 12 well-plates were allowed to recover the mechanical stress for about 48 hours after slicing and treated with 1% ODE or 10 ppm H_2_S (by adding NaSH) or ODE+H_2_S for 6 hours daily for 5 days. Following 6 hours of exposure to treatments, treatment media was replaced with culture medium after washing with DPBS. In a separate set, both NHBE cells and murine PCLS were pre-treated either with vehicle control (DMSO) or RTA-408 (20 ng or 100 ng) respectively for two hours/day for a total of five days followed by the exposure to either medium or ODE or H_2_S daily for six hours/day for a total of five days. The 6-hour cell culture supernatants were collected each day and stored at -80°C until processed. The cell pellets and lung slices were collected after 5-day treatment and either processed for RNA and protein extraction or stored in -80°C.

**Table 1:** Experimental design.

	NHBE cells	PCLS
Treatment 1 Dose	RTA-408 20 ng/mL	RTA-408 100 ng/mL
Treatment 2 Dose	Control/ODE/H_2_S/ODE+ H_2_S ODE (0.5%) H_2_S (10 ppm)	Control/ODE/H_2_S/ODE+ H_2_S ODE (1%) H_2_S (10 ppm)

### Quantification of Altered Cell Morphology

After the completion of the 5-day treatment, the altered morphology of NHBE cells was quantified. Majority of the cells in the microscopic fields were touching the neighboring cells and hence a computer software (ImageJ) based quantification of length and area of the cells was deemed not very accurate. Therefore, after the images were captured, an experienced researcher blinded to the treatment groups manually counted the cells in 20X microscopic fields (n=4-6/group) which were enlarged or had altered shape and or vacuolation or invagination of the nucleus. All the cellular changes documented were with respect to the basal control as a reference. The percent of cells with altered morphology was statistically analyzed and graphically represented.

### Quantification of Cell Viability

We performed an MTT assay to quantify the cell metabolic activity to quantify the viability of the NHBE cells and PCLS after treatments. About 2×10^4^ NHBE cells/well were seeded in a 96-well cell culture plate and incubated in a humidified chamber under a 5% CO_2_ incubator until the cells reached 70–80% confluence and individual murine PCLS were placed in 96-well cell culture plate with similar conditions. After the completion of treatments in both NHBE cells and PCLS, MTT dye ((3-(4,5-dimethylthiazol-2-yl)-2,5-diphenyltetrazolium bromide) (Invitrogen) was loaded (5 ng/mL) into each well-containing the cells and the murine PCLS and incubated at 37°C for another 3-5 h. At the end of the incubation period, the supernatant was discarded and 100 µL of DMSO (Sigma) was added into each well and dye absorbance (450 nm) of each of the 96-wells was read using SpectraMax M2 Gemini Microplate Reader (Molecular Devices, San Jose, CA). Basal control cells were used as a baseline and the absorbance values of all the groups were normalized and expressed as % viability.

### *Klebsiella pneumoniae* Culture and Infection

*Klebsiella pneumoniae* (ATCC 43816, American Type Culture Collection, Manassas, MA) strain was used in this study. The bacterium was cultured in Luria-Bertani (LB) broth and LB agar. The Multiplicity of Infection (MOI) of 1:100 was used for NHBE cells, whereas for every slice of the murine lungs (PCLS), 10^5^ CFUs (Colony Forming Units) was added to infect. After the completion of 5-day treatment either medium or ODE or H_2_S or ODE+H_2_S, NHBE and murine PCLS were respectively inoculated with a fresh bacterial culture. We allowed a bacterial contact time of 2 hours for NHBE cells and 4 hours for PCLS before performing an invasion assay.

### Invasion Assay

The invasion assay was performed as per the published protocols (Cortés et al., 2002; Banerjee et al., 2019) to quantify the number of bacteria entering the cells or lung tissues. Briefly, in NHBE cells, the infection was followed by incubation of cells with gentamycin (100 mg/mL) for 2 hours and then the cells were lysed with 0.02% SDS and plated in LB agar at different dilutions. After the infection, lung slices were incubated with gentamycin (100 mg/mL) for 2 hours followed by digestion of the tissue in digestion solution (1.5 mg/mL collagenase+ 0.4 mg/mL DNase 1, 10 mM HEPES and 5% FBS) and plated on LB agar at different dilutions. The inoculated plates were incubated aerobically at 37°C for 24 hours and the colonies were counted manually for each treatment group. The number of CFUs was enumerated, data was normalized over control group and graphically presented.

### Immunofluorescence Staining

NHBE cells grown on poly-D-lysine (PDL) coated glass coverslips were exposed either to medium (control) or 0.5% ODE or H_2_S (10 ppm) or ODE+ H_2_S for 6 hours daily as described above and processed for immunofluorescence staining. Briefly, the cells were fixed with 4% ice cold paraformaldehyde for 20 minutes and blocked by incubating in the 10% donkey serum for an hour at the room temperature. Next, the cells were stained with primary antibodies against NRF2 (1:400), 4-HNE (1:500), 3-NT (1:500) Occludin (1:500), Ezrin (1:500), and E-cadherin (1:500) overnight at 4° C. Anti-E-cadherin was procured from Cell Signaling Technologies, 4-HNE and 3-NT from Abcam and all other antibodies were obtained from the Santa Cruz Biotechnologies. Following overnight incubation, coverslips were incubated with an anti-species biotinylated secondary antibody (Jackson ImmunoResearch, 1:300) for an hour and streptavidin cy3 (Jackson Immunoresearch Laboratories Inc., West Grove, PA, 1:400) for 30 minutes at room temperature. Five randomly chosen fields per slide (1 slide/group) were captured through a camera attached to the microscope (Nikon Eclipse TE2000-U inverted fluorescence microscope Nikon, Tokyo, Japan) and processed using the ImageJ program (National Institute of Health) to measure the staining intensity and mean intensity (Cy3) per cell was calculated using HCImageLive software and represented graphically. To avoid overlapping in image acquisition and counting the cells in the same area twice, we followed a particular zig-zag pattern of imaging. We started from one corner and moved in a zig-zag pattern until we reached the other corner of the slide area. In addition, we used computer software-based image intensity analysis and researchers blinded to the treatment groups to avoid a potential bias.

### Western Blotting

The cell pellets and lung tissue homogenates were lysed in RIPA buffer with a 1X protease inhibitor cocktail (Thermo Fisher Scientific, USA) and the resulting homogenates were centrifuged at 14000 rpm for 20 minutes at 4°C for whole-cell or tissue lysate preparation. Protein concentration was determined using a Bradford protein assay kit (Bio-Rad, USA). Equal amounts of protein were separated using different gel percentages; 7.5%, 10%, and 12% SDS-PAGE gels and electro transferred onto nitrocellulose membranes overnight using a constant voltage (23V). Next, the membranes were incubated with the fluorescent blocking buffer (Rockland Immunochemicals, PA, USA) in PBS with 0.05% Tween-20 for an hour at room temperature to reduce non-specific binding followed by incubation with primary antibodies for staining overnight at 4°C. Primary antibodies included mouse anti-NRF2 (1:1000, Santacruz) and rabbit anti-β-actin (1:10000, ab8227). The blots were washed in 1X PBS with 0.1% tween 20 (PBST) for 15-20 minutes twice and stained with secondary antibodies consisting of Alexa Fluor 680 Donkey anti-Rabbit IgG (1:10000, Jackson) or anti-mouse 680 Alexa Fluor antibodies (1:10000, Thermo Fisher Scientific). Membranes were scanned using the Odyssey^®^ CLx IR imaging system (LI-COR Biotechnology, Lincoln, NE). The band intensity (densitometry analysis) values were calculated using ImageJ (National Institute of Health) and band intensity for protein of interest was normalized twice. At first, β-actin (housekeeping gene) band intensities were normalized with respect to the basal controls. Next, values for band intensity of the proteins of interest were normalized with respect to their respective control and then with respect to respective β-actin values. These steps ensured that the variation in the intensities of the bands for housekeeping protein (β-actin) did not introduce any bias.

### Quantitative Real-Time PCR (qRT-PCR)

The cell pellets and lung tissue homogenates were digested in TRIZOL (Sigma) for RNA extraction. The RNA concentration was measured using a microplate reader (SpectraMax M2 Gemini, Molecular Devices, San Jose, CA). Total RNA (0.5-2 µg) was used for the cDNA synthesis using the Superscript III first-strand synthesis kit (Thermo Fisher Scientific, USA) as per the manufacturer’s protocol. For the qRT-PCR, 1 µL of cDNA per 10 µL of reaction volume including 1 µL of primers each, 5 µL of SYBR green and nuclease free water were used. Reactions were performed using forward and reverse primers. Sequences of all the primers (forward and reverse) listed in **Table 2** were synthesized at Iowa State University’s DNA Facility. The housekeeping gene 18 S rRNA (Thermo Fisher Scientific, USA) was used in all the qPCR reactions. No template and no-primer controls and dissociation curves were run for all the reactions to exclude cross-contamination. The qRT-PCR reactions were run in a QuantiStudio 3 system (ThermoFisher Scientific) and the data was analyzed using 2^-ΔΔCT^ method (Livak and Schmittgen, 2001). The data was normalized over the housekeeping gene 18S and control and represented as bar graphs.

**Table 2:** Human primers.

Human gene of interest	Primers	(5’→3)
*nrf2*	Forward	CACATCCAGTCAGAAACCAGTGG
	Reverse	GGAATGTCTGCGCCAAAAGCTG
*nqo1*	Forward	CCTGCCATTCTGAAAGGCTGGT
	Reverse	GTGGTGATGGAAAGCACTGCCT
*hox1*	Forward	CCAGGCAGAGAATGCTGAGTTC
	Reverse	AAGACTGGGCTCTCCTTGTTGC
*gclc*	Forward	GGAAGTGGATGTGGACACCAGA
	Reverse	GCTTGTAGTCAGGATGGTTTGCG
*gclm*	Forward	TCTTGCCTCCTGCTGTGTGATG
	Reverse	TTGGAAACTTGCTTCAGAAAGCAG
*ecadherin*	Forward	GCCTCCTGAAAAGAGAGTGGAAG
	Reverse	TGGCAGTGTCTCTCCAAATCCG
*occludin*	Forward	ATGGCAAAGTGAATGACAAGCGG
	Reverse	CTGTAACGAGGCTGCCTGAAGT
*ezrin*	Forward	ATCGAGGTGCAGCAGATGAAGG
	Reverse	CGCAGCATCAACTCCTCCTTCT
*mlck2*	Forward	GCTGTATGCAGCCATCGAGACT
	Reverse	ATGGTGTCCACCTCGGTCAGAT
*keap1*	Forward	CAACTTCGCTGAGCAGATTGGC
	Reverse	TGATGAGGGTCACCAGTTGGCA
**Mouse primers**		
**Mouse gene of interest**	**Primers**	**(5’→3’)**
*nrf2*	Forward	CAGCATAGAGCAGGACATGGAG
	Reverse	GAACAGCGGTAGTATCAGCCAG
*nqo1*	Forward	GCCGAACACAAGAAGCTGGAAG
	Reverse	GGCAAATCCTGCTACGAGCACT
*hox1*	Forward	CACTCTGGAGATGACACCTGAG
	Reverse	GTGTTCCTCTGTCAGCATCACC
*gclc*	Forward	ACACCTGGATGATGCCAACGAG
	Reverse	CCTCCATTGGTCGGAACTCTAC
*gclm*	Forward	TCCTGCTGTGTGATGCCACCAG
	Reverse	GCTTCCTGGAAACTTGCCTCAG
*ecadherin*	Forward	GGTCATCAGTGTGCTCACCTCT
	Reverse	GCTGTTGTGCTCAAGCCTTCAC
*occludin*	Forward	TGGCAAGCGATCATACCCAGAG
	Reverse	CTGCCTGAAGTCATCCACACTC
*ezrin*	Forward	GATGCCCAAGCCAATCAACG
	Reverse	AAGGGAAGAAGATCTTTGGG
*claudin3*	Forward	TCATCGTGGTGTCCATCCTGCT
	Reverse	AGAGCCGCCAACAGGAAAAGCA
*claudin5*	Forward	TGACTGCCTTCCTGGACCACAA
	Reverse	CATACACCTTGCACTGCATGTGC
*claudin18*	Forward	GTCACGACGTAGCCAGAATACC
	Reverse	CCATCCGAAAAAGTAGGACCAGG
*mlck2*	Forward	TACGCAGCCATTGAGACCTCTC
	Reverse	ATGGTGTCCACCTCCGTCAGAT
*Keap1*	Forward	ATCCAGAGAGGAATGAGTGGCG
	Reverse	TCAACTGGTCCTGCCCATCGTA

### Cytokine Assay

Cytokines were quantified using commercially available kits (Thermo Fisher Scientific) following the manufacturer’s instructions. The lower limit of detection for all the cytokines was 2 pg/mL. Briefly, 96-well high binding plates (Nunc MaxiSorp, ThermoFisher Scientific) were coated with a capture antibody (100 µL/well) and incubated at 4°C overnight. Wells were blocked with a blocking buffer (200 µL/well) for an hour at room temperature followed by washing with 1X PBST (three times) and then, incubated with 100 µL/well of recombinant standards or samples in duplicate wells (cell culture supernatants) for two hours at room temperature. Next, plates were washed thrice with PBST and incubated with a detection antibody (100 µL/well) for an hour at room temperature. Finally, incubation was performed with 3,3’,5,5’-Tetramethylbenzidine (TMB) solution (100 µL/well) for 15 minutes (in dark) after removal of the detection antibody and washing 5-7 times. The color development reaction was stopped by adding a stop solution (2N H_2_SO_4,_ 50 µL/well). The absorbance was read at 450 nm using SpectraMax M2 Gemini Microplate Reader (Molecular Devices, San Jose, CA).

### Measurement of Transepithelial Electrical Resistance (TEER)

NHBE cells were seeded in collagen coated 0.4 µm cell culture inserts with the expansion media in both the upper and lower compartments. Once the cells reached confluence, the media in the upper chamber was removed and the media in the lower chamber was changed to differentiation media and cells were allowed to differentiate for another 25-28 days. Next, the various treatments were applied (as described above in the experimental design. The trans-epithelial electrical resistance (TEER, ohm) was measured using epithelial voltammeter with silver chloride “chopstick” electrodes (World Precision Instruments). Prior to measurement, the apical layer of the air-liquid interface (ALI) cultures was washed with 1X DPBS and 1X DPBS was added to both apical and basolateral sides to equilibrate the cultures. The resistance obtained from a cell-free culture insert was subtracted from the resistance measured across each cell monolayer and the TEER values of epithelial cells were expressed in Ω cm^2^ after correcting for the surface area of the culture insert.

### Fluorescent Dextran (Permeability) Assay

Permeability of the cellular monolayer was assessed by measuring the movement of the apically applied FITC-dextran across the cellular monolayer (Smyth et al., 2020). NHBE cells were seeded in collagen coated 3 µm cell culture inserts with the expansion media in both the upper and lower compartment. Once the cells reached confluence, the media in the lower compartment (basal side) was changed to differentiation media and the media in the upper compartment (apical side) was removed and cells were allowed to polarize at the air-liquid interface. Next, various treatments (as described in the treatment protocol above in the experimental design) were applied in the basal compartment with media and dextran-fluorescein (MW 3,000) was added apically for one hour after the completion of the last treatment. Fluorescence (excitation 485/emission 535) in the basal compartment was measured using microplate reader (SpectraMax M2 Gemini, Molecular Devices, San Jose, CA) and the values were graphically represented.

### Statistical Analysis

Data was analyzed and graphically represented using GraphPad Prism 8.0 software (La Jolla, CA, USA). Raw data were analyzed either using one-way ANOVA or two-way ANOVA followed by the Tukey’s *post-hoc* test to compare all the treatment groups. A p-value of ≤ 0.05 was considered statistically significant. An * indicates significantly different from control and # indicates a significant difference between basal and RTA-408 treatment group (*/# p < 0.05, **/## p < 0.01, ***/### p < 0.001, ****/#### p < 0.0001).

## Results

### Nrf2 Activation With RTA-408 Reverses ODE+H_2_S Induced Morphological Changes in NHBE Cells

**Figure 1:**
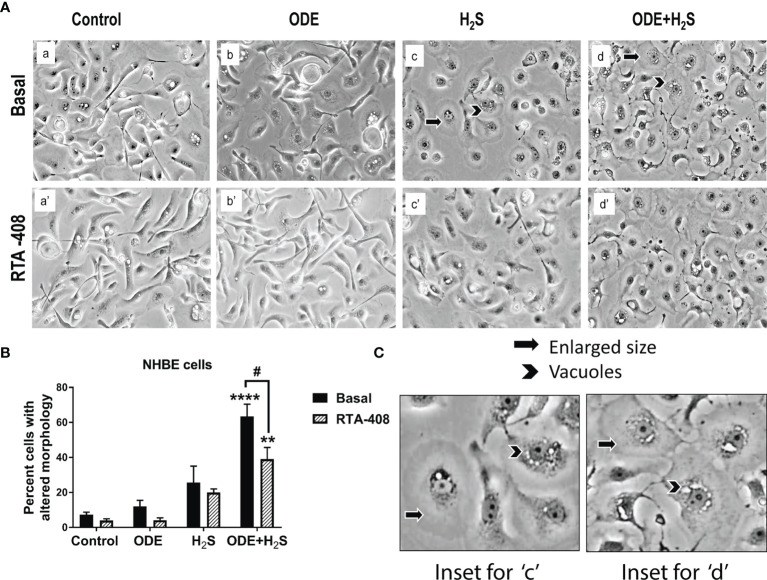
RTA-408 (Nrf2 activator) improved the cell morphology. Phase contrast microscopic images (20X) were captured after the completion of the exposure protocol for 5 days with or without RTA-408 (20ng pre-exposure for 2 hours) treatment followed by exposure to media (control) or ODE (0.5%) or H_2_S (10ppm) or ODE+H_2_S. Repeated exposure to ODE+H_2_S co-exposure caused the increase in the size of the cells **(A–C)** as compared to controls. Whereas no significant increase in cell size was seen for those treated with ODE or H_2_S alone as compared to controls. Also, nuclear vacuolization was more prominent in ODE+H_2_S co-exposure groups. Pre-exposure to RTA-408 improved the cell size and lowered the nuclear vacuolization in ODE+H_2_S co-exposure group as compared to those without RTA-408 treatment Data (mean ± SEM) analyzed with two-way ANOVA followed by Tukey’s *post hoc* test is shown (n=4-6/group) and p ≤ 0.05 was considered significant (* compared to basal control, # between basal and RTA-408).

Morphological changes indicative of cellular stress was quantified by counting the cells that showed enlargement and or vacuoles. Repeated exposure of NHBE cells to ODE or H_2_S or ODE+H_2_S distorted the cell morphology (**Figure 1**). The cells exposed to ODE+H_2_S showed significantly altered morphology (enlarged as compared to the basal control cells and were round in comparison to the elongated cells) when compared to the basal control group (**Figures 1A, B**). Also, cells exposed to ODE, H_2_S and ODE+H_2_S without the RTA treatment showed a prominent nuclear vacuolization when compared to the basal controls. However, RTA-408 pre-treatment (Nrf2 activator) in ODE+H_2_S significantly reversed the cell morphology changes (**Figures 1A, B**). Additionally, the degree of nuclear vacuolization was decreased with RTA-408 pre-treatment in ODE+ H_2_S group when compared to its respective basal group.

### Repeated Exposure to ODE and H_2_S Decreases the Viability of the NHBE Cells and Murine PCLS

**Figure 2:**
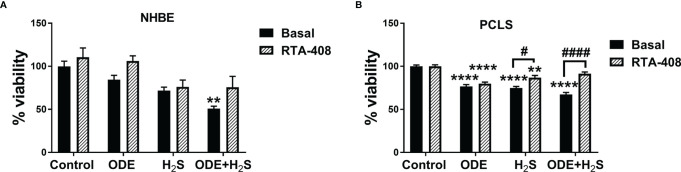
Repeated exposure to H_2_S and H_2_S with ODE diminished metabolic activity of cells. MTT assay was performed to assess the viability of NHBE cells and PCLS after the repeated exposure. Results show that the NHBE cell viability is significantly decreased with ODE+H_2_S treatment as compared to the basal control **(A)**. Similarly, the viability of PCLS significantly decreased with repeated exposure to ODE, H_2_S and ODE+H_2_S as compared to basal control **(B)**. Pre-treatment with RTA-408 significantly improved the viability of PCLS in H_2_S and ODE+H_2_S as compared to their respective basal groups **(B)**. Data (mean ± SEM) analyzed with two-way ANOVA followed by Tukey’s *post hoc* test is shown (n=5/group) and p ≤ 0.05 was considered significant (* compared to basal control, # between basal and RTA-408).

Repeated exposure to ODE, H_2_S and ODE+H_2_S significantly decreased the viability of the NHBE cells and PCLS (**Figures 2A, B**). In NHBE cells, ODE+H_2_S exposure significantly decreased the viability as compared to the media exposed controls (**Figure 2A**). Similarly, there were decreases in viability in PCLS model as compared to basal controls with exposure to ODE, H_2_S, and ODE+H_2_S. Moreover, RTA-408 pre-treatment of PCLS resulted in increased viability following exposure to H_2_S and ODE+H_2_S but no significant change was demonstrated with ODE exposure (**Figure 2B**). Further, RTA-408 pre-treatment did not change the viability in NHBE cells.

### Repeated Exposure to ODE and H_2_S Decreases the Nrf2 Expression in NHBE Cells and PCLS *via* Enhanced Keap1 Expression

**Figure 3:**
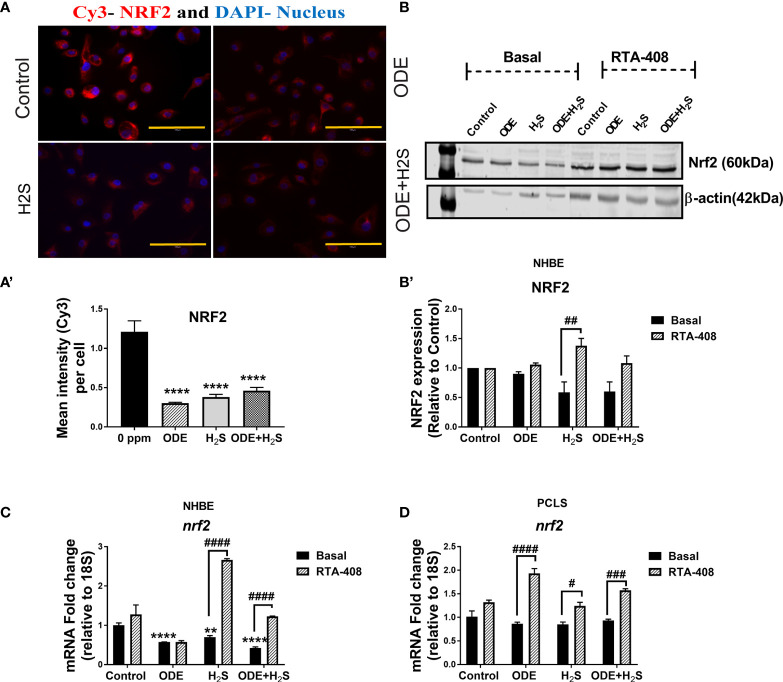
Repeated exposure to ODE and H_2_S decreased the Nrf2 expression. Immunofluorescence microscopy (40X) **(A)**, western blotting **(B-B’)** and RT-qPCR **(C, D)** were performed to quantify the expression of Nrf2 with repeated exposure to ODE and H_2_S in NHBE **(A–C)** and PCLS **(D)**. Immunofluorescence microscopy and RT-qPCR showed decreased Nrf2 (Red) expression **(A, C)**. Mean intensity of NRF2 staining (per 5-8 fields) was calculated to quantify the expression **(A’)**. Densitometry values (n=4/group) normalized over housekeeping proteins (β-actin) and NRF2 band intensities (relative to control) are shown (b-b’). RTA-408 pre-treatment increased the expression of Nrf2 in H_2_S and ODE+H_2_S group as compared to respective basal group **(B-B’)**. Also, RTA-408 pre-treatment in NHBE **(C)** and PCLS **(D)** showed increased folds of *nrf2* transcript as compared to the respective basal groups. Data (mean ± SEM) analyzed with two-way ANOVA followed by Tukey’s *post hoc* test is shown and p ≤ 0.05 was considered significant (* compared to basal control, # between basal and RTA-408).

**Figure 4:**
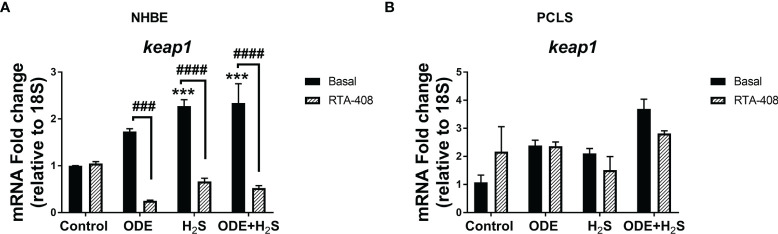
Repeated exposure H_2_S or ODE+H_2_S increased the Keap1 transcript in NHBE cells. Keap1 mRNA was quantified using Rt-qPCR in both NHBE cells **(A)** and PCLS **(B)** after the repeated exposure with and without pre-exposure to RTA-408. Keap1 mRNA transcript significantly increased with H_2_S and ODE+H_2_S as compared to basal control in NHBE cells while RTA-408 treatment decreased the Kepa1 mRNA levels significantly **(A)**. Data (mean ± SEM) analyzed with two-way ANOVA followed by Tukey’s *post hoc* test is shown and p ≤ 0.05 was considered significant (* compared to basal control, # between basal and RTA-408).

Repeated exposure of NHBE cells to ODE, H_2_S and ODE+H_2_S diminished the protein and gene expression of Nrf2 (**Figures 3A, C**). This decreased Nrf2 expression was accompanied by an increase in Keap 1 gene expression in NHBE cells (**Figure 4A**). However, pre-treatment with RTA-408 significantly increased the expression of Nrf2 protein in H_2_S group. RTA-408 treatment did not increase Nrf2 expression in ODE alone or H_2_S+ODE as compared to H_2_S basal group (**Figures 3B, B’**). When compared to the respective basal controls, Nrf2 mRNA fold change was significantly increased in RTA-408 pre-treatment in H_2_S and ODE+H_2_S groups but not in ODE alone group (**Figure 3C**). Correspondingly, RTA-408 pretreatment in NHBE cells significantly diminished the fold change in the expression of Keap1 mRNA in ODE, H_2_S and ODE+H_2_S as compared to the respective basal groups (**Figure 4A**). Similarly, RTA-408 pre-treatment significantly increased Nrf2 transcripts in ODE, H_2_S and ODE+ H_2_S groups when compared to their respective basal groups in the murine PCLS model (**Figure 3D**). However, there was no change in Keap1 gene expression with any of the exposures (ODE, H_2_S, and ODE+ H_2_S) and there was no change with RTA-408 pre-treatment (**Figure 4B**) in PCLS model.

### Nrf2 Activation With RTA-408 Rescues ODE and H_2_S Exposure Induced Suppression of Nrf2 Mediated Antioxidants in NHBE Cells and PCLS

**Figure 5:**
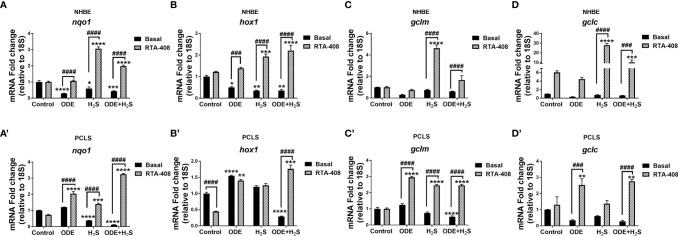
RTA-408 increased the expression of downstream genes of NRF2 antioxidant pathway. Nrf2 antioxidant downstream genes, *nqo1* **(A-A’)**, *hox1* **(B-B’)**, *gclm* **(C-C’)** and *gclc* **(D-D’)** were quantified using Rt-qPCR in both NHBE cells **(A–D)** and PCLS (A’-D’) after the repeated exposure to ODE or H_2_S or ODE+H_2_S with and without pre-exposure to RTA-408. Nrf2 downstream gene expression quantified by RT-qPCR were normalized over media exposed cells (basal control). Repeated exposure to ODE, H_2_S and ODE+ H_2_S decreased the gene expression of *nqo1* (A-A’), *hox1* (B-B’) as compared to basal control. Pre-treatment with RTA-408 significantly increased the mRNA transcript of *nqo1* **(A-A’)**, *hox1* **(B-B’)**, *gclm* **(C-C’)** and *gclc* **(D-D’)** after the repeated exposure to ODE or H_2_S or ODE+H_2_S. Data (mean ± SEM) analyzed with two-way ANOVA followed by Tukey’s *post hoc* test is shown and p ≤ 0.05 was considered significant (* compared to basal control, # between basal and RTA-408).

Downstream expression of antioxidant genes of the Nrf2 pathway was measured following repeated exposure to ODE, H_2_S, and ODE+H_2_S versus controls in NHBE cells (**Figures 5A-D**) and PCLS (**Figures 5A’–D’**) models respectively. Repeated exposure to ODE, H_2_S, and ODE+H_2_S significantly decreased *nqo1* and *hox1* transcripts in NHBE cells when compared to basal controls (**Figures 5A, B**). In PCLS, H_2_S and ODE+H_2_S treatment significantly decreased *nqo1* gene transcripts (**Figure 5A’**) and ODE+H_2_S exposure significantly decreased *hox1* and *gclm* gene transcripts (**Figures 5B’, C’**) when compared to the basal control. However, pre-treatment with RTA-408 of ODE, H_2_S and ODE+H_2_S significantly increased the gene expression of *nqo1* (**Figures 5A, A’**), *hox1*, *gclm* (**Figures 5C, C’**) and *gclc* (**Figures 5D, D’**) when compared to their respective basal groups in both NHBE cells and PCLS and when compared to basal controls as indicated.

### Repeated Exposure to ODE and H_2_S Increases the Expression of 4-HNE and 3-NT and Nrf2 Activation With RTA-408 Decreases the Expression of 3-NT in NHBE Cells Exposed to ODE or H_2_S

**Figure 6:**
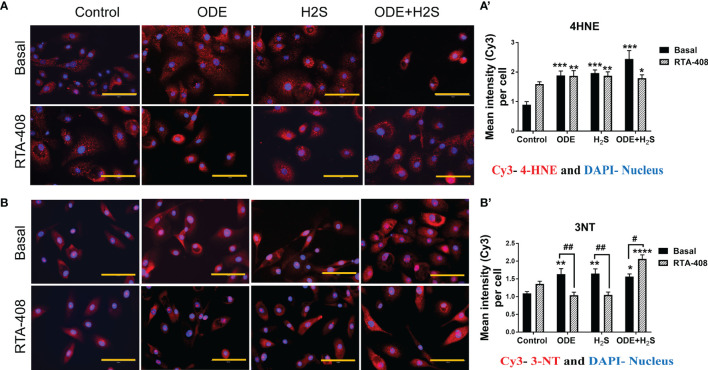
RTA-408 treatment decreased the oxidative stress. Immunofluorescence staining (40X) was performed to quantify the oxidative stress markers, 4-hydroxynonenal (4-HNE) **(A)** and 3-nitrotyrosine (3NT) **(B)**. Mean intensities of 3-NT and 4-HNE (per 5-10 fields) were calculated to quantify the expression **(A’, B’)**. Both 3-NT (Red) and 4-HNE staining (Red) was increased with repeated exposure to ODE or H_2_S or ODE+H_2_S **(A’-A’, B-B’)**. Whereas RTA-408 pre-treatment decreased 3-NT expression **(B-B’)**. Data (mean ± SEM) analyzed with two-way ANOVA followed by Tukey’s *post hoc* test is shown and p ≤ 0.05 was considered significant (* compared to basal control, # between basal and RTA-408).

In these studies, expression of 4-hydroxynonenal (4-HNE) and 3-nitrotyrosine (3-NT) to represent lipid peroxidation and protein nitration as representative oxidative stress markers were assessed (**Figure 6**). Based on the quantitative microscopy data, repeated exposure to ODE, H_2_S and ODE+H_2_S in NHBE cells increased 4-HNE and 3-NT expression by immunofluorescence staining intensity (**Figures 6A, B**). This observed increase was confirmed by quantification (**Figures 6A’, B’**). Moreover, RTA-408 pre-treatment decreased the 3-NT expression in ODE and H_2_S treated groups as compared to their respective basal groups (**Figures 6B, B’**). In contrast, RTA-408 pre-treatment increased 3-NT expression in cells exposed to ODE+H_2_S when compared to the respective basal controls (**Figures 6B, B’**). RTA-408 pre-treatment did not change the expression of 4-HNE from any of the treatment groups (ODE, H_2_S and ODE+H_2_S) (**Figures 6A, A’**). Additionally, the expression was still significantly higher when compared to basal control.

### Activation of Nrf2 With RTA-408 Reduces Levels of IL-6 and IL-1β Induced by Repeated Exposure to ODE and H_2_S in NHBE Cells and PCLS

**Figure 7:**
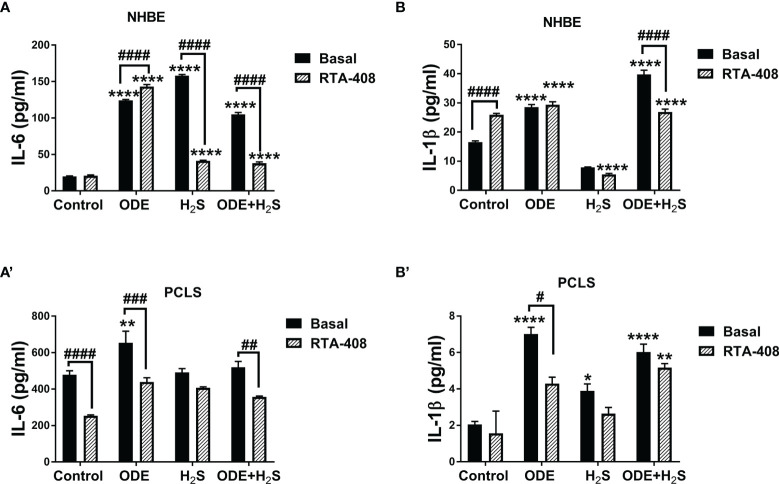
RTA-408 treatment decreased inflammatory cytokines. Using ELISA, inflammatory cytokines, IL-6 and IL-β secreted in the supernatants of both NHBE cells **(A, B)** and PCLS **(A’, B’)** were quantified after the repeated exposure to ODE or H_2_S alone or ODE+H_2_S co-exposure. Repeated exposure to ODE and H_2_S elevated the inflammatory cytokines; IL-6 **(A-A’)** and IL-β **(B-B’)**. Pre-treatment with RTA-408 significantly decreased the secretion of IL-6 **(A-A’)** and IL-β **(B-B’)**. Data (mean ± SEM) analyzed with two-way ANOVA followed by Tukey’s *post hoc* test is shown and p ≤ 0.05 was considered significant (* compared to basal control, # between basal and RTA-408).

Repeated exposure to ODE, H_2_S and ODE+H_2_S in both NHBE cells (**Figures 7A, B**) and PCLS (**Figures 7A’, B’**) significantly increased IL-6 and IL-1β levels measured in cell/tissue-free supernatant when compared to the basal control. RTA-408 pre-treatment of cells significantly decreased IL-6 secretion in H_2_S and ODE+ H_2_S treated groups in NHBE (**Figure 7A**) and in ODE and ODE+ H_2_S treated groups in PCLS model (**Figure 7A’**) when compared to respective basal groups. Similarly, RTA-408 pre-treatment significantly decreased IL-1β secretion in ODE+ H_2_S treated group in NHBE (**Figure 7B**) and with ODE alone in the PCLS model (**Figure 7B’**). However, IL-6 and IL-1β secretion was still higher when compared to basal control. However, RTA-408 pre-treatment significantly increased the IL-6 levels following ODE alone exposure and IL-1β levels in the basal controls when compared to their respective controls in the NHBE cells (**Figures 7A, B**).

### Repeated Exposure to ODE and H_2_S Altered the Epithelial Barrier Function and Nrf2 Activation With RTA-408 Rescued the Epithelial Barrier Integrity in NHBE Cells

**Figure 8:**
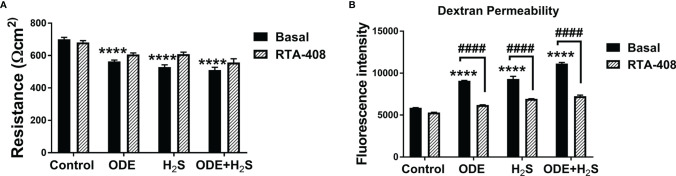
RTA-408 improved the transepithelial permeability. Transepithelial electrical resistance (TEER) **(A)** and Dextran permeability assay **(B)** (n=5-6/group) were performed to measure the effect of repeated exposure to ODE, H_2_S and ODE+H_2_S on Normal human bronchial epithelial (NHBE) cells grown on Air-Liquid interface (ALI). Repeated exposure to ODE or H_2_S or ODE+H_2_S significantly decreased the transepithelial electrical resistance as compared to media exposed control **(A)** and at the same time, significantly increased the transepithelial permeability **(B)**. Pre-treatment with RTA-408 improved transepithelial permeability in all the groups as compared to ones without RTA-408 **(B)**. Data (mean ± SEM) analyzed with two-way ANOVA followed by Tukey’s *post hoc* test is shown and p ≤ 0.05 was considered significant (* compared to basal control, # between basal and RTA-408).

**Figure 9:**
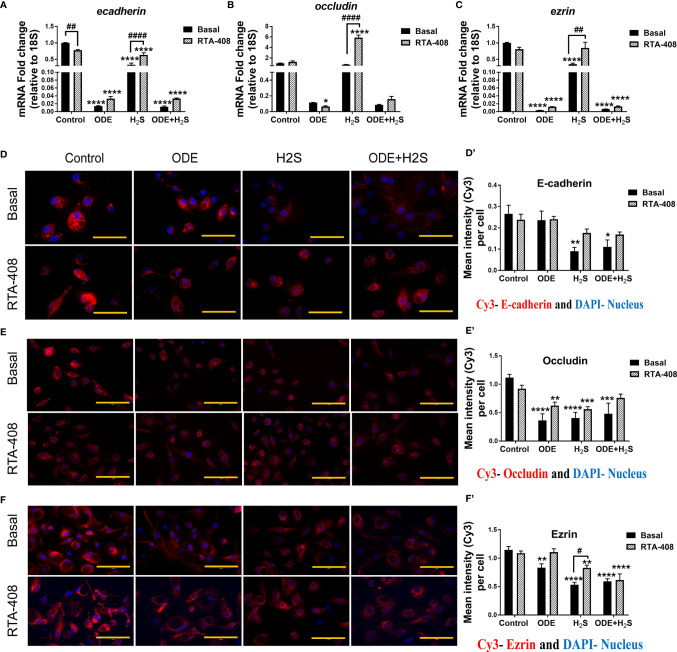
RTA-408 improved the tight junction protein expression. RT-qPCR (n=3/group) was used to measure the fold change in tight junction mRNAs **(A–C)** in NHBE cells grown in ALI. Repeated exposure to ODE, H_2_S and ODE+H_2_S significantly decreased the folds of *ecadherin* **(A)** and *ezrin* **(C)** transcripts. Pre-treatment with RTA-408 improved the tight junction mRNA expression in H_2_S exposed group as compared to H_2_S basal group **(A–C)**. Immunofluorescence staining (40X) (n=5-10 fields/group) in submerged NHBE cells showed decreased tight junction protein expression, E-cadherin (Red) **(D-D’)**, Occludin (Red) **(E-E’)** and Ezrin (Red) **(F-F’)**. RTA-408 pre-treatment in H_2_S exposed group increased the ezrin expression as compared to basal H_2_S **(F-F’)**. Data (mean ± SEM) analyzed with two-way ANOVA followed by Tukey’s *post hoc* test is shown and p ≤ 0.05 was considered significant (* compared to basal control, # between basal and RTA-408).

We measured the epithelial barrier integrity in ALI cultures by using transepithelial electrical resistance (TEER) and fluorescent dextran permeability assay. Repeated exposure to ODE, H_2_S and ODE+H_2_S in ALI cultures significantly decreased the TEER values (**Figure 8A**) with a corresponding increase in permeability (**Figure 8B**). RTA-408 pre-treatment of cells exposed to H_2_S significantly increased the TEER values (**Figure 8A**). Permeability effects were partially reversed with Nrf2 activation with RTA-408 (**Figure 8B**). Next, we measured mRNA transcripts corresponding to tight junction proteins in ALI cultures exposed to ODE, H_2_S and ODE+H_2_S with or without RTA-408 pre-treatment (**Figures 9A–C**). Repeated exposure to ODE, H_2_S and ODE+H_2_S in ALI cultures significantly decreased the *ecadherin* and *ezrin* mRNA transcripts as compared to the respective basal controls (**Figures 9A, C**). Whereas occludin expression did not significantly change with different treatments (**Figure 9B**). However, RTA-408 pre-treatment in H_2_S group significantly increased *ecadherin, occludin* and *ezrin* mRNA transcripts when compared to basal H_2_S group (**Figures 9C–E**). Repeated exposure to ODE, H_2_S and ODE+H_2_S significantly decreased the immunofluorescence staining intensity of E-cadherin (**Figures 9D, D’**), occludin (**Figures 9E, E’**) and ezrin (**Figures 9F, F’**) in NHBE cells. However, RTA-408 pre-treatment significantly increased the ezrin expression in H_2_S group as compared to H_2_S basal group (**Figures 9F, F’**).

### Repeated Exposure to ODE and H_2_S Altered the Tight Junction mRNA Transcripts and Nrf2 Activation With RTA-408 Pretreatment Rescued Their Expression in PCLS

**Figure 10:**
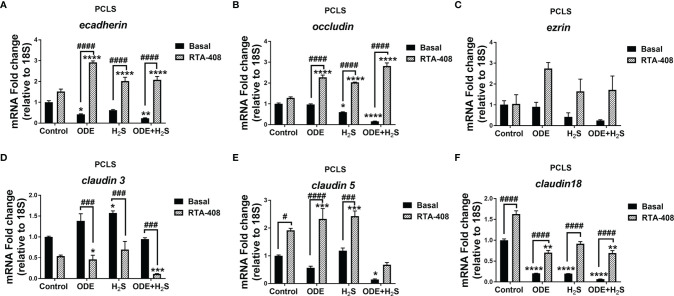
RTA-408 treatment altered the expression of tight junction proteins in PCLS. RT-qPCR (n=3/group) was performed to measure the fold change in tight junction mRNAs **(A–F)** in PCLS). Repeated exposure to ODE, H_2_S and ODE+H_2_S in PCLS significantly decreased the folds of *ecadherin* **(A)**, *occludin* **(B)**, *claudin 5* **(E)** and *claudin 18* **(F)** transcripts and significantly increased *claudin 3 transcript* **(D*)*** as compared to basal control. While the RTA-408 pre-treatment significantly increased the folds of tight junction transcripts as compared to their respective basal groups; *ecadherin* **(A)**, *occludin* **(B)**, *claudin 5* **(E)** and *claudin 18* **(F)**. *claudin 3* mRNA fold change was significantly decreased with RTA-408 pre-treatment as compared to their respective basal groups **(D)**. Data (mean ± SEM) analyzed with two-way ANOVA followed by Tukey’s *post hoc* test is shown and p ≤ 0.05 was considered significant (* compared to basal control, # between basal and RTA-408).

Repeated exposure to ODE, H_2_S and ODE+H_2_S in PCLS altered the tight junction mRNA transcripts (**Figure 10**). Repeated exposure in different treatment groups reduced the gene expression of *ecadherin* (**Figure 10A**)*, occludin* (**Figure 10B**)*, claudin5* (**Figure 10E**) *and claudin18* (**Figure 10F**). Expression of *ecadherin*, and *claudin18* but not *claudin5* was reduced with ODE alone. H_2_S treatment reduced *occludin* and *claudin18* expression when compared to basal control. In contrast, only H_2_S significantly increased *claudin3* mRNA when compared to basal control (**Figure 10D**). RTA-408 pre-treatment significantly increased *ecadherin, occludin, claudin5* and *claudin18* mRNA transcripts but decreased *claudin3* mRNA transcript in various groups when compared to their respective basal groups. However, repeated exposure in different treatment groups with or without RTA-408 had no effect on *ezrin* mRNA expression (**Figure 10C**).

### Repeated Exposure to ODE and H_2_S Increased the Bacterial Invasion in Both NHBE Cells and PCLS

**Figure 11:**
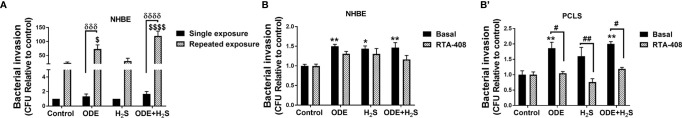
Repeated exposure increased the bacterial (*K. pneumoniae*) invasion. Invasion assay was performed to enumerate the number of bacteria (*K. pneumoniae*) entering the NHBE cells (n=3-8/group) and PCLS (n=4/group) after the completion of the treatment protocol. Repeated exposure to ODE and ODE+H_2_S significantly increased the bacterial load in NHBE cells as compared to single exposure for 6 hours **(A)**. Repeated exposure to ODE, H_2_S and ODE+H_2_S in NHBE cells **(B)** and PCLS (B’) significantly increased the bacterial invasion as compared to basal control. RTA-408 pre-treatment significantly decreased the bacterial invasion in PCLS as compared to their respective basal groups. Data (mean ± SEM) analyzed with two-way ANOVA followed by Tukey’s *post hoc* test is shown and p ≤ 0.05 was considered significant. ($ compared to repeated exposure control, δ between single and repeated exposure * compared to basal control, # between basal and RTA-408). */# p < 0.05, **/## p < 0.01, ***/###/δδδ p < 0.001, ****/####/δδδδ p < 0.0001.

*Klebsiella pneumoniae* invasion was increased in airway epithelial cells repeatedly exposed (5 days) to ODE and ODE+H_2_S as compared to cell exposed once to ODE or ODE+H_2_S (**Figure 11A**). Similarly, repeated exposure to ODE, H_2_S and ODE+H_2_S in NHBE cells and ODE and ODE+H_2_S (in PCLS resulted in increased *Klebsiella pneumoniae* invasion when compared to the basal control (**Figures 11B, B’**). RTA-408 pre-treatment had no effect on the bacterial invasion in NHBE cells (**Figure 11B**). However, in PCLS subjected to repeated exposures, Nrf2 activation with RTA-408 significantly reduced bacterial invasion in ODE, H_2_S and ODE+H_2_S exposed groups as compared to their respective basal groups (**Figure 11B’**).

### Nrf2 Activation With RTA-408 Decreased the (nm)MLCK Transcript in NHBE Cells and PCLS

**Figure 12:**
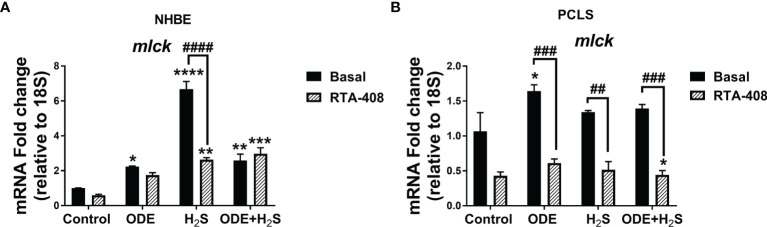
RTA-408 treatment decreased the MLCK transcript. RT-qPCR (n=3/group) was performed to measure the fold change in mlck mRNAs (a-f) in NHBE cells **(A)** and PCLS **(B)**. Repeated exposure to ODE or H_2_S or ODE+H_2_S in NHBE cells significantly increased the folds of *mlck* transcripts **(A)** and ODE significantly increased the folds of mlck transcript as compared to basal control in PCLS model **(B)**. RTA-408 pre-treatment significantly decreased the folds of mlck transcripts in H_2_S group as compared to basal group in NHBE **(A)**. RTA-408 pre-treatment significantly decreased the folds of mlck transcripts when compared to respective basal groups in PCLS **(B)**. Data (mean ± SEM) analyzed with two-way ANOVA followed by Tukey’s *post hoc* test is shown and p ≤ 0.05 was considered significant (* compared to basal control, # between basal and RTA-408).

Repeated exposure to ODE, H_2_S and ODE+H_2_S in NHBE cells (**Figure 12A**) and ODE in PCLS (**Figure 12B**) significantly increased the non-muscle *mlck* (myosin light chain kinase, an enzyme that plays a role in maintaining epithelial tight junction proteins) mRNA transcript when compared to basal control group. However, RTA-408 pre-treatment in H_2_S group in NHBE cells significantly decreased non-muscle *mlck* mRNA transcript when compared to H_2_S basal group (**Figure 12A**). Similarly, in PCLS, RTA-408 pre-treatment in ODE, H_2_S and ODE+H_2_S groups significantly decreased non-muscle *mlck* mRNA transcript when compared to respective basal groups (**Figure 12B**).

## Discussion

The current study demonstrates that repeated exposure to ODE alone, H_2_S alone, and ODE+H_2_S increases the cellular oxidative stress with the concomitant reduction in epithelial barrier integrity. This disruption of epithelial barrier integrity was associated with an increase in bacterial invasion. As we confirmed the previous work that endogenous Nrf2 expression was increased with exposures, we further sought to define the role of Nrf2 with pharmacologic activation. Indeed, RTA-408 treatment increased the Nrf2 expression and resulted in the rescue of the oxidative stress-induced loss of epithelial barrier integrity. This was collectively demonstrated by a decrease in the bacterial invasion and decreased production of inflammatory cytokines. Together, these studies strongly support targeting Nrf2 activation to potentially reduce adverse oxidative stress outcomes induced by ODE or H_2_S exposure, ultimately decreasing the occupational exposure induced respiratory disease burden.

While repeated exposure to ODE alone, H_2_S and ODE+H_2_S induced overwhelming oxidative damage and loss of epithelial barrier function, the combined (ODE+H_2_S) exposure failed to elicit a full synergistic effect. Barring a synergistic effect induced in altering cellular morphology and decrease in the viability in NHBE cells, ODE+H_2_S exposure did not induce an exaggerated inflammation or cellular damage in both models. This could be due to reaching the maximal inflammatory potential of the exposures in our models. The other possible effect could be that of possible anti-inflammatory effect due to H_2_S (Gemici and Wallace, 2015). Our previous work (Shrestha et al., 2021) has shown that a single exposure of human monocytic (THP-1) and bronchial epithelial (BEAS-2B) cell lines to H_2_S induced significant production of IL-10, an anti-inflammatory cytokine. It is interesting to note that, despite a possible anti-inflammatory effect of exposure to H_2_S, the combined exposure to ODE+H_2_S induced significant cellular damage and loss of barrier function to indicate translational relevance to worker’s health.

Under hemostasis, Nrf2 expression is maintained at a basal level and is continuously targeted by Keap1 and rendered inactive by 26S proteosome-mediated degradation. When there is oxidative stress, Nrf2 escapes degradation, enters nucleus to induce the transcription of antioxidant genes (Lo et al., 2006; Rada et al., 2012). However, chronic respiratory diseases such as COPD are known to cause a reduction in the levels and activity of Nrf2 (Sussan et al., 2009; Harvey et al., 2011; Yamada et al., 2016). In the present study, repeated exposure to ODE or H_2_S alone or ODE+H_2_S decreased the expression of Nrf2 in NHBE cells and a reduced transcription of downstream cytoprotective genes, NQO1, HO-1 and GCLM to indicate cellular oxidative stress. Our quantitative analysis of morphological changes in cells revealed that, compared to controls, ODE and H_2_S groups, exposure to ODE+H_2_S resulted in a significantly higher percentage of cells with enlarged size and nuclear vacuolation. It is possible that some of these atomic vacuolations could be nuclear membrane invaginations. Nevertheless, such changes usually indicate underlying cellular stress (Bavle, 2016). Despite decreased Nrf2 expression (immunofluorescence data) in ODE, H_2_S and ODE+H_2_S exposed cells; significant morphological changes were evident only in the ODE+H_2_S group. Further, RTA-408 mediated activation of Nrf2 rescued the pathologic cellular changes to indicate the importance of Nrf2 mediated antioxidant responses (Yamamoto et al., 2018).

We observed differences in how human primary NHBE cells and mouse PCLS responded to the exposure to ODE alone or H_2_S alone and ODE+H_2_S. These differences were marked in repeated exposures to the above contaminants. This phenomenon could be explained based on the following facts. The murine PCLS represent a physiologically relevant lung model with the architecture of cells as in an *in vivo* model due to the structural arrangement of epithelial cells (type I and type II), endothelial cells, fibroblasts, and interstitial macrophages, dendritic cells, T cells. Further, in addition to cell-to-cell interaction, PCLS exhibit cilia beating, contraction of airway smooth muscle and mucus production similar to the lungs functioning *in vivo* (Lyons-Cohen et al., 2017; Liu et al., 2019). These factors may explain why RTA-408 mediated Nrf2 activation may have a synergistic effect while acting on multiple cell types and hence the therapeutic effects are pronounced in PCLS model when compared to the NHBE cells. On the contrary, RTA-408 reduces *keap1* mRNA levels significantly in NHBE cells but not in the PCLS model. The most plausible explanation for this difference would be the observed differences in the basal expression of keap1 and Nrf2 in these two models as well as anatomical and functional complexity (as explained above) of the PCLS. When we pre-treated NHBE cells or PCLS with RTA-408, it increased the Nrf2 levels in H_2_S exposed NHBE cells (both mRNA and protein) and all three exposures of PCLS model (protein data) along with an increased expression of the downstream target genes indicating the therapeutic potential of RTA-408 mediated activation of Nrf2 pathway. Our observations are in line with the findings of McGovern et al., who reported that sulforaphane (Nrf2 activator) pre-treatment in OD exposed BEAS-2B cells had higher expression of *nqo1, hox1* as compared to sulforaphane pre-treated controls (McGovern et al., 2019). He et al., explains how Nrf2 mediated upregulation of the cytoprotective genes reduce reactive nitrogen species (3NT) levels (He and Ma, 2012).

Our five-day exposure models (NHBE cells and PCLS) mimic the oxidative stress observed in similar exposure models (Natarajan et al., 2016; Bhat et al., 2019). Our results showing elevated levels of 3NT and 4HNE indicate oxidative stress along with a loss of Nrf2 activity. Others have shown that activation of Nrf2 abrogates oxidative stress (Sussan et al., 2009; Miller et al., 2013; Li et al., 2016) and experimental deletion of Nrf2 in mice is associated with increased oxidative stress (Delgado-Buenrostro et al., 2015), underscoring the importance of exposure-induced loss of Nrf2. Our findings link the exposure-induced increase in Keap1 mRNA levels in NHBE cells as a possible mechanism to explain the reduction in Nrf2 following exposures to the contaminants. Further, our results support the hypothesis that Nrf2 activation is a potential therapeutic target.

Our IL-1β and IL-6 data indicate that exposure to ODE, H_2_S and ODE+H_2_S induce oxidative stress as well as an increase in the production of inflammatory cytokines. RTA-408 mediated activation of Nrf2 activation showed partial protection by decreasing IL-1β and IL-6 in both NHBE cells and PCLS models. It is possible that exposure-induced disrupted Nrf2 activity might be one of the potential reasons for an increase in IL-1β and IL-6 secretions. A previous finding that Nrf2 provides cryoprotection by inhibiting the transcription of the inflammatory genes supports this explanation. Nrf2 inhibits the transcription of the proinflammatory cytokines TNFα, IL-1β, IL-6 by targeting the NF-кB pathway since the NF-кB pathway activation is enhanced in Nrf2 deficient mice as compared to the wild type mice (Thimmulappa et al., 2006; Michaličková et al., 2020). Furthermore, it is demonstrated that NF-кB mediated transcription of inflammatory cytokines is downregulated by activation of Nrf2-antioxidant signaling pathways by regulating the IкBк degradation (Thimmulappa et al., 2006; Li et al., 2008). Thus, our data establish the benefits of targeting Nrf2 by showing reductions in key inflammatory cytokines.

Oxidative stress is potentially one of the mechanisms by which disruption of the epithelial barrier occurs following exposure to several pollutants ranging from particulate matters to infectious biologicals (Heijink et al., 2012; Tharakan et al., 2016). We observed a significant decrease in TEER accompanied by significant increase in paracellular permeability. Furthermore, the expression of tight junction proteins and mRNA transcripts significantly decreased with repeated exposure to contaminants in both NHBE cells and PCLS. In both of these models, the expression of *e-cadherin*, *occludin* and *ezrin* mRNA transcripts was decreased as compared to basal control. *Claudin 5* and *claudin 18* mRNA transcripts decreased significantly in PCLS. Conversely, *claudin 3* mRNA transcripts significantly increased in PCLS model. These data mirror a previous study in which an increased expression of *claudin 3* resulted in increased permeability (Mitchell et al., 2011; Cuzić et al., 2012). This observation could be explained by the fact that increased levels of claudin 3 is associated with increased malignancy in few clinical cases (Zhang et al., 2017), possibly explaining its role in chronic inflammation.

Decreased expression of most of the tight junction proteins analyzed coincided with the increase in oxidative stress and elevated secretion of inflammatory cytokines. The exact mechanism of the oxidative stress induced disruption of epithelial barrier integrity is not explored in our current study. Though our work does not uncover underlying mechanisms of oxidative stress and how it may be disrupting the airway epithelial barrier, other studies have shown how oxidative stress can alter the epithelial tight junctions by derangement of actin cytoskeleton, disruption of adheres junctions and post-translational protein modifications caused by thiol oxidation, phosphorylation, nitration and carbonylation (Rao, 2008). Our results show that Nrf2 activation partially rescued epithelial barrier function in NHBE cells. ODE exposure-induced barrier disruption could be explained by the fact that organic dust consists of proteases (Romberger et al., 2015) that induce the proteolytic breakdown of cellular cytoskeleton which could be synergistic to the damage due to oxidative stress. Nevertheless, the advantages of activation of the Nrf2 are supported by the rescue of the epithelial barrier dysfunction in asthma models and cigarette smoke-induced epithelial disruption and subsequent improvement of the pulmonary dysfunction (Sussan et al., 2015; Tharakan et al., 2016).

The chief function of the tight junction proteins is to maintain the barrier integrity of the epithelial cells to forbid the entry of any foreign elements into the respiratory system. The importance of barrier function is underscored by an increase in the incidences of bacterial and viral infections in people who have occupational exposure to chemicals, particulate matter and organic dust (Torén et al., 2020). Our study reports an increased invasion of the *K. pneumoniae* in NHBE cells and PCLS exposed to ODE, H_2_S and ODE+ H_2_S compared to the untreated ones. A previous study has shown that exposure to particulate matter increased the *Pseudomonas aeruginosa* invasion by elevating oxidative stress (Liu et al., 2020). In addition to this, another study demonstrated that such exposures increased the virulence of *Pseudomonas aeruginosa* (Mushtaq et al., 2013). In NHBE cells, Nrf2 activation with RTA-408 did not help to minimize the bacterial invasion. Our data could explain this phenomenon because Nrf2 activation only partially rescued the tight junction proteins along with the TEER values. Whereas, in the PCLS model, Nrf2 activation with RTA-408 diminished the bacterial invasion. This might be due to the presence of other defense mechanisms such as the presence of macrophages, functional cilia and mucus. Further, Nrf2 activation decreased the oxidative stress that improved the epithelial barrier function and decreased the bacterial invasion in PCLS. Thus, NHBE cells and PCLS models show distinct responses to ODE exposure induced epithelial barrier disruption, Nrf2 activation and bacterial invasion. These dichotomous results underscore the importance of physiologically relevant models such as PCLS. Our study is limited by the use of cells and PCLS but not an animal model such as a mouse. Future studies using a mouse model of exposure would be valuable.

The non-muscle myosin light chain kinase (nmMLCK) is activated upon the oxidative stress and plays a vital role in the regulation of the barrier function by interacting with the perijuctional actomyosin ring (PAMR) (Usatyuk et al., 2012; Wang et al., 2018; He et al., 2020). In our study, nmMLCK mRNA transcript expression increased following exposure to all three contaminants, and nmMCLK expression decreased with Nrf2 activation. Previous findings have shown that Nrf2 inhibits the MLCK activation by negatively regulating its transcription (Liu et al., 2018). Thus, inhibition of nmMLCK activity is possibly one of the potential mechanisms of how Nrf2 might regulate the tight junction barrier integrity and minimize exposure-induced oxidative stress. These mechanistic data will be helpful in developing better therapeutic agents against exposure-induced loss of barrier function. Further, polymorphism in the Nrf2 gene is associated with susceptibility to cancer (Hartikainen et al., 2012). Therefore, examining whether farm workers have a polymorphism in this gene would be beneficial.

## Conclusion

We demonstrated that exposure to ODE and H_2_S increased oxidative stress, produced inflammatory cytokines and resulted in the loss of epithelial tight junction proteins and barrier integrity. These exposures relevant to agriculture environments increased *K. pneumoniae* invasion in human airway epithelial cells and murine lung slices. Findings were also associated with increased levels of Keap1 and decreased expression of Nrf2, and importantly, pharmacologic activation of Nrf2 rescued the vast majority of exposure induced changes, including bacterial invasion in the murine lung slice model. Future *in vivo* studies targeting Nrf2 pathway would bring a translational relevance to the current findings. Lastly, it would be interesting to examine whether barn workers experience loss of Nrf2 activity in their respiratory tract as well as if there is a polymorphism of *Nrf2* gene in the farm workers (Cho et al., 2015).

## Data Availability Statement

The raw data supporting the conclusions of this article will be made available by the authors, without undue reservation.

## Ethics Statement

The animal study was reviewed and approved by Institutional Animal Care and Use Committee (IACUC), Iowa State University, Ames, IA, USA.

## Author Contributions

DS participated in the design of experiments, performed the experiments, analyzed the data, and drafted the manuscript. NM performed organic dust extraction. SB assisted in culturing of NHBE cells. OS and QZ provided the technical expertise required to perform bacterial culture and participated in editing the manuscript. TJ provided the technical expertise required to perform fluorescent microscopy and participated in editing the manuscript. KB supplied NHBE cells and technical advice in culturing the cells and edited the manuscript. JP provided technical expertise in designing exposure models and participated in editing the manuscript. CC conceptualized the study, participated in the design of the experiments, performed dust extraction, participated in the interpretation of data, and edited the manuscript. All authors have read and approved the final manuscript.

## Funding

CC laboratory is funded through startup grant through Iowa State University, a pilot grant (5 U54 OH007548) from CDC-NIOSH (Centers for Disease Control and Prevention-The National Institute for Occupational Safety and Health) and a seed grant though CVM (College of Veterinary Medicine) at the Iowa State University. JP is supported by grants from the National Institute for Occupational Safety and health (R01OH012045 and U54OH010162), Department of Defense (PR200793) and the Central States Center for Agricultural Safety and health (CS-CASH). JP has also received funding from AstraZeneca and Takeda for projects unrelated to this current work.

## Conflict of Interest

The authors declare that the research was conducted in the absence of any commercial or financial relationships that could be construed as a potential conflict of interest.

## Publisher’s Note

All claims expressed in this article are solely those of the authors and do not necessarily represent those of their affiliated organizations, or those of the publisher, the editors and the reviewers. Any product that may be evaluated in this article, or claim that may be made by its manufacturer, is not guaranteed or endorsed by the publisher.

## Abbrevaition

OD: Organic Dust; ODE: Organic Dust Extract; H2S: Hydrogen sulfide; Nrf2: Nuclear factor-erythroid factor 2-related factor 2; NHBE: Normal Human Bronchial Epithelial ; ALI: Air Liquid Interface; PCLS: Precision Cut Lung Slice Culture; Keap 1: Kelch Like ECH Associated Protein 1; NQO1: NAD(P)H Quinone Dehydrogenase 1; HOX1: Heme oxygenase 1: GCLC: Glutamate-Cysteine Ligase Catalytic Subunit; GCLM: Glutamate-Cysteine Ligase Modifier Subunit; NaSH: Sodium Hydosulfide; 4-HNE: 4-hydroxynonenal; and 3-NT:3-nitrotyrosine; DMSO: Dimethyl Sulfoxide.
